# Combined clinical and research training in medical physics in a multi‐institutional setting: 13‐year experience of Harvard Medical Physics Residency Program

**DOI:** 10.1002/acm2.13806

**Published:** 2022-11-08

**Authors:** Yulia Lyatskaya, Brian Winey, W. S. Kiger, Martina Hurwitz, Piotr Zygmanski, G. Mike Makrigiorgos, Thomas R. Bortfeld, Karen P. Doppke, Xing‐Qi Lu, Lee M. Chin, Peter Biggs, David P. Gierga

**Affiliations:** ^1^ Department of Radiation Oncology Brigham and Women's Hospital Boston Massachusetts USA; ^2^ Department of Radiation Oncology Massachusetts General Hospital Boston Massachusetts USA; ^3^ Department of Radiation Oncology Beth Israel Deaconess Medical Center Boston Massachusetts USA; ^4^ Harvard Medical School Boston Massachusetts USA

**Keywords:** medical physics, medical physics residency

## Abstract

**Purpose:**

This manuscript describes the structure, management and outcomes of a multi‐institutional clinical and research medical physics residency program (Harvard Medical Physics Residency Program, or HMPRP) to provide potentially useful information to the centers considering a multi‐institutional approach for their training programs.

**Methods:**

Data from the program documents and public records was used to describe HMPRP and obtain statistics about participating faculty, enrolled residents, and graduates. Challenges associated with forming and managing a multi‐institutional program and developed solutions for effective coordination between several clinical centers are described.

**Results:**

HMPRP was formed in 2009 and was accredited by the Commission on Accreditation of Medical Physics Education Programs (CAMPEP) in 2011. It is a 3‐year therapy program, with a dedicated year of research and the 2 years of clinical training at three academic hospitals. A CAMPEP‐accredited Certificate Program is embedded in HMPRP to allow enrolled residents to complete a formal didactic training in medical physics if necessary. The clinical training covers the material required by CAMPEP. In addition, training in protons, CyberKnife, MR‐linac, and at network locations is included. The clinical training and academic record of the residents is outstanding. All graduates have found employment within clinical medical physics, mostly at large academic centers and graduates had a 100% pass rate at the oral American Board of Radiology exams. On average, three manuscripts per resident are published during residency, and multiple abstracts are presented at conferences.

**Conclusions:**

A multi‐institutional medical physics residency program can be successfully formed and managed. With a collaborative administrative structure, the program creates an environment for high‐quality clinical training of the residents and high productivity in research. The main advantage of such program is access to a wide variety of resources. The main challenge is creating a structure for efficient management of multiple resources at different locations. This report may provide valuable information to centers considering starting a multi‐institutional residency program.

## 1 INTRODUCTION

Medical physics is a highly specialized field that requires many years of education and training.^1^, ^2^ Demand for medical physicists has been high and is projected to continue to grow.^3^ Although there are different career paths leading to a career in medical physics,^4^ completion of a medical physics residency accredited by the Commission on Accreditation of Medical Physics Education Programs (CAMPEP)^5^ has become a requirement to be certified by the American Board of Radiology (ABR)^6^ and work as an independent clinical medical physicist.^7^ As of May 2022, there are 152 CAMPEP‐accredited medical physics residency programs, of which 113 programs are accredited in therapy.^5^ The curriculum and requirements for the medical physics residencies are formalized in several documents, including American Association of Physics in Medicine (AAPM) Task Group reports^8^ and CAMPEP standards.^9^ The basic elements of the curriculum for the therapy track include Treatment Equipment, Detectors and Dosimetry, Treatment Planning, Imaging, Brachytherapy, Radiation Safety, Professionalism and Ethics, and Informatics. In addition, there are several elective modules that are not required but may offer a benefit to the residents in providing the breadth of knowledge and flexibility in their job search, although some may only be available at selected centers.^8^ For residency programs, the decision to offer elective modules is most likely based on the available resources. Therefore, some programs may choose to focus on in‐depth coverage of the required material, whereas other programs could add optional topics, especially if the clinical facility associated with the residency has the equipment and expertise to provide this training. To augment potentially limited in‐house clinical experience, a “hub and spoke” model may also be utilized, where the hub provides administrative oversight to the residency program, whereas spokes can complement clinical opportunities not available at hub.^10^ To the best of our knowledge, few residencies follow this structure,^11^, ^12^, ^13^ and no publications exist on their experience managing such programs.

Offering research opportunities within the residencies may also be challenging due to the requirement that 24 full months be devoted to clinical training.^9^ To overcome this limitation, some residencies, mostly within large academic centers, extend the duration of the residency to 3 or even 4 years. As of May 2022, 12 programs in therapy have a residency duration greater than 24 months.^5^

Although the hub and spoke structure may help to expand clinical resources available to the residents, a multi‐institutional program may be beneficial in offering breadth in both research and clinical training, providing the residents access to a wide variety of clinical resources, as well as research laboratories. However, covering a wide range of topics on varied equipment requires extra time and creates a burden on the faculty and residents. Therefore, it is of interest to investigate if forming such a program is viable and beneficial.

In this report, we describe our experience creating and managing the Harvard Medical Physics Residency Program (HMPRP).^14^ To the best of our knowledge, this is the only CAMPEP‐accredited multi‐institutional medical physics residency program with combined clinical and research training. The program was formed in 2009 and achieved CAMPEP accreditation in 2011. It has been reaccredited twice since then. We aim to share our experience of creating this residency program more than 10 years ago and management strategies for its successful operation to help other residencies decide if a multi‐institutional approach may be beneficial for their training programs.

## 2 METHODS

In this section we describe the history, structure, and governance of HMPRP, highlighting its perceived benefits. In addition, we describe the metrics used to characterize the program's success. We also describe the perceived and encountered challenges and utilized solutions when forming HMPRP.

### 2.1 History of the program

HMPRP was formed as a combined program between three academic hospitals located in close geographical proximity to each other. The hospitals have well‐established Radiation Oncology Departments that are associated with a single Medical School and have employed and trained medical physicists over several decades, although no formal training program existed at the time. The discussions on the creation of a medical physics residency program among these academic hospitals initiated in 2007, and the program was formed in 2009. It received full CAMPEP accreditation in 2011 and two subsequent reaccreditations, one in 2015 and again in 2021. In addition, the program was approved by the graduate medical education (GME) committees of the respective hospitals. It is of note that a multi‐institutional program for *medical residents* shared by these three hospitals (Harvard Radiation Oncology Program) has been in existence since 2002, and the goal of our medical physics residency program was to build on experience and follow the success of the medical residency program.

### 2.2 Objectives of the program

The main objective of the program is to provide comprehensive training and experience in radiation oncology physics to the enrolled residents and ultimately educate the next generation of medical physicists, researchers, and leaders. With this goal, our program aims to attract outstanding candidates, including those with accredited medical physics graduate degrees as well as those from outside the field. To accommodate candidates who need to take didactic courses to satisfy the CAMPEP course requirement,^15^ a decision was made to offer the didactic courses within the residency program. These courses were later grouped under a single Certificate Program that received CAMPEP accreditation in 2015. It should be noted that our program can accommodate a Certificate Program within our residency only because it is a 3‐year residency.

### 2.3 Anticipated challenges of HMPRP

The multi‐institutional nature of the program presents unique challenges for its management. The main challenges that were anticipated at the time of the program creation are summarized as follows:

1. Ensuring administrative and financial support from all participating institutions. Lack of such support may affect the availability of faculty and the number of hired residents per year.
2. Facilitating frequent and effective communication between members of the program leadership based at different institutions. Although the institutions are close geographically, they are located on different campuses in an urban environment, and commuting makes frequent in‐person communication challenging, requiring a comprehensive multilayered leadership structure.
3. Developing an advanced yet realistic curriculum. Without careful coordination of resources and scheduling of clinical rotations, the training curriculum covering a wide variety of equipment is too intense and unwieldy.
4. Coordination of multiple faculty in the resident training. Without proper coordination, effective communication, and oversight, many participating faculty may create the potential for scheduling conflicts, gaps in curriculum flow, redundancy, and overlap of covered material.
5. Monitoring residents’ progress in a complicated structure involving many faculty and residents residing at different institutions may be especially challenging without well‐organized feedback mechanisms for both the faculty and the residents.

In the following sections, we describe several approaches developed in our program to help mitigate the challenges outlined above.

### 2.4 Multi‐institutional program structure and governance

As the first step in creating a multi‐institutional program, support from the administration of all three institutions was solicited and received, both from the Department Chairs and Department Administrative Directors. This support is critical to ensure the allocation of faculty time for teaching and access of residents to training resources (treatment machines and equipment). However, financial support of the residents was considered separately. Only two institutions have committed at this time to provide salary to the residents, whereas the third institution provides faculty time allocation and resources only. Therefore, two residents are hired per year in our program, one resident to be supported by each financially contributing institution; however, both residents receive clinical training in all three institutions.

**FIGURE 1:**
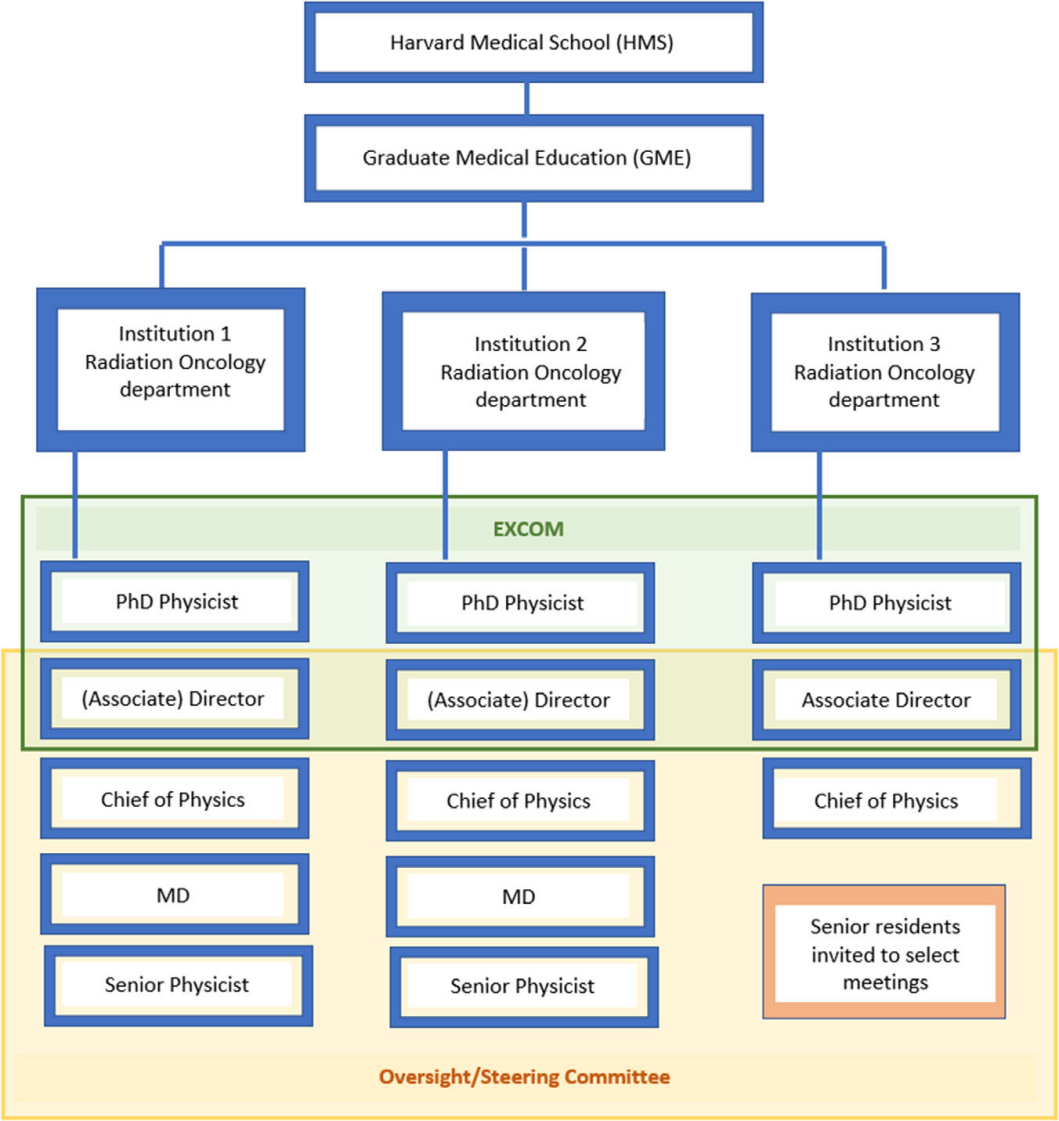
Schematic diagram of the governance structure of Harvard Medical Physics Residency Program (HMPRP)

To provide continuous operation and a solid oversight of the program, a comprehensive leadership structure with representatives from the three institutions was created, illustrated in Figure 1.

#### 2.4.1 Program Director and Associate Program Directors

A sole Program Director is required to be responsible and accountable for ensuring that the residency program satisfies the CAMPEP standards.^9^ However, the function of a Program Director may differ in a multi‐institutional setting compared to a residency formed within a single institution. Many administrative decisions, including institutional support, financial support of the residents, allocating faculty time for teaching, securing access of the residents to the resources within individual hospitals, communication with the faculty and staff at individual institutions, etc., can be better managed by a person affiliated with that particular institution. To mitigate these challenges, the positions of Associate Program Directors were created, while preserving a role of a sole Program Director. At any given time, a representative of one of the two financially supporting institutions serves in the role of Program Director, whereas representatives from other institutions are serving in the roles of Associate Programs Directors. The (sole) Program Director rotates every 3 years between the institutions.

#### 2.4.2 The Executive Committee (EXCOM)

To provide support and serve as a backup for the Program Director and the Associate Program Directors, an additional person is selected from each of the three institutions. These six individuals form the Program's Executive Committee (EXCOM) that is responsible for the operation, management, and continuous improvement of the training program. EXCOM meets on a monthly basis throughout the year and more frequently at the time of the candidate selection.

#### 2.4.3 The Steering/Oversight Committee

In addition to EXCOM, a Steering Committee (initially, an Oversight Committee) was formed to provide higher level support to the Program. The Steering Committee consists of the Program Director, the two Associate Program Directors, the Chiefs of the Medical Physics Divisions from the three hospitals, two attending physicians (from different institutions), and two additional physicists. As physicians bring a unique and complementary perspective to the committee, the commitment to attend the committee meetings and the residency interviews are important considerations for the candidates for this role. Familiarity with the medical residency management is an additional advantage, but not a requirement. The additional physicists are selected from those who are not directly involved in the management of the residency program on a day‐to‐day basis to help focus on a big picture perspective. Senior residents are invited to provide feedback on behalf of all residents prior to each Steering Committee meeting. The Steering Committee is co‐chaired by the Program Director and one of the Chiefs of the Medical Physics Divisions from a different institution than the Program Director. The Committee meets semi‐annually.

#### 2.4.4 Senior Resident

In 2017, the role of Senior Resident in HMPRP was introduced to provide guidance and leadership at a peer level for all residents enrolled in the program and to help coordinate various activities between residents in different years of enrollment. The initial reason for establishing a formal senior resident role was to provide a mentoring resource for newer residents; however, it was later recognized that this role also provides an opportunity to develop leadership skills. This duty is performed by the two third‐year residents at their respective locations.

### 2.5 Multi‐institutional approach to curriculum implementation

In the following sections, we describe the multi‐institutional approach to curriculum implementation, the goals for the research year, the schedule and details of clinical rotations, and various approaches to creating the feedback mechanisms. Although more detailed information is available on the program's website^14^ and in the program's Handbook,^16^ in this section, we summarize the details of curriculum that reflect the current state of the program as well as specific reasons for the current rotation schedule.

#### 2.5.1 First year: research

Each year, EXCOM collects from the faculty multiple clinically relevant projects that have a reasonable chance of substantial completion within one year. These projects are offered to the incoming residents for review. Each resident selects one of the projects and starts their research overseen by a mentor who monitors the progress of the resident throughout the year.

One reason for designating the first year for research is that candidates who enter the program without a background in medical physics can take the necessary didactic courses to meet CAMPEP and ABR requirements through the embedded CAMPEP‐accredited Certificate Program. The course schedule in our program is designed to allow a resident to take the courses without violating the CAMPEP requirement to take no more than two courses during the clinical years.^9^ Another reason for research within the first year is to provide the residents with an opportunity to continue a (limited) research trajectory throughout residency. Although the residents are expected to complete the first manuscript by the end of the first year, in case they have enough data for another publication, it can be finalized with minimal time commitment over the following year. In addition, research tools that the residents build working on their research project may be applicable to smaller clinical projects that the residents work on during their clinical rotations thus expanding the resident's exploration of the medical physics topics.

#### 2.5.2 Years two and three: clinical training

In Table 1, the schedule of clinical rotations in the year 2021 is presented together with assignments of the mentors from different institutions. The main goal in structuring our curriculum in this specific way is to allow the residents to cover all included material in the most efficient way, while rotating between the institutions. The main challenges and approaches to solve them are described as follows:

1. The main challenge of designing a curriculum with a wide range of material to cover is allocating sufficient time for each module. Running the modules sequentially presented a practical challenge as every module would be too short to allow the residents sufficient opportunities to observe the procedures that are not frequently scheduled. For this reason, the decision was made to run several modules in parallel. The modules that can be successfully covered simultaneously are Treatment Planning, which can be performed mostly during the day, Treatment Equipment and Dosimetry with hands‐on practice after hours, and Radiation Safety that requires multiple observations of procedures performed infrequently (e.g., source swap, shipment of radioactive materials, etc.). This model was implemented for the rotations in the first clinical year of training. In the second clinical year, the modules are more focused, and we found that it was more productive to run most of these modules sequentially.
2. The second challenge was to find the right duration for each rotation. In the first years of the residency's existence, it was recognized that the residents need more time in the very first rotation to become familiar with the clinic and master the basics. For this reason, the first rotation was extended from 6 to 7 months, and the second rotation was shortened from 6 to 5 months. The modules in the second clinical year are 2–3 months in duration, which we found sufficient.
3. Another challenge was to avoid redundancy when residents rotate between institutions. Based on the feedback from the residents and the results of the oral assessments, the curriculum in the first clinical year is designed to cover same modules in two different institutions with minimal overlap. Thus, a single curriculum is followed, whereas practical tasks are performed on different equipment in different institutions. For example, linac quality assurance (QA) is performed on Varian linacs in Institution 1 and on Elekta linacs in Institution 2, although specific QA topics are discussed in depth at one institution only. To allow for efficient integration of residents in a new clinic, they undergo a one‐week orientation to the clinic at the start of the first clinical year, and when rotating from Institution 1 to Institution 2. In the second clinical year, the modules are shorter and more focused, typically located at a single institution.
4. Allocating time for educational conferences and seminars may also be challenging. In addition to the research, didactic course, and clinical rotation requirements, the residents are expected to attend clinical conferences, seminars, lectures, meetings that are relevant to their training and contribute to their continuing education, and present at journal clubs and departmental physics seminars. Each resident is also required to attend three workshops on ethics or professionalism per year and is expected to present at national and local chapter meetings. As these didactic sessions are an integral part of the resident's training, the attendance requirements as well as formal time allocation for these activities are important.

**TABLE 1:** Schedule of clinical rotations

Clinical year 1				
Treatment planning	7 months		Institution 1	Mentor 1
Treatment equipment	7 months	Modules run in parallel	Institution 1	Mentor 2
Dosimetry	7 months		Institution 1	Mentor 2
Radiation safety	7 months		Institution 1	Mentor 3
Treatment planning	5 months		Institution 2	Mentor 4
Treatment equipment	5 months	Modules run in parallel	Institution 2	Mentor 5
Dosimetry	5 months		Institution 2	Mentor 5
Radiation safety	5 months		Institution 2	Mentor 6

Clinical year 2				
Brachytherapy	2 months		Institution 1	Mentor 7
Stereotactic—conventional linac	6 weeks	Modules run in parallel	Institution 1	Mentor 8
MR‐linac	1 week		Institution 1	Mentor 9
Stereotactic—CyberKnife	4 weeks		Institution 3	Mentor 10
Imaging and informatics	2 weeks		Institution 3	Mentor 11
Training at network locations	1 month	Modules run in parallel	Satellite clinic—Institutions 1 or 2	Mentor 12
Protons	2 months		Institution 2	Mentor 13
Brachytherapy	4 months		Institution 2	Mentor 14
Treatment planning	4 months		Institution 2	Mentor 4
Nuclear medicine	1 week		Institution 2	Mentor 15
Education and professionalism	12 months	Module runs in parallel with all other modules	Institutions 1 and 2	Program Director and Associate Program Director

### 2.6 Multi‐institutional resources

#### 2.6.1 Faculty

The biggest asset of HMPRP is the multi‐institutional faculty, spanning both the clinical and research aspects of medical physics.

*Clinical faculty*: For each training module, a resident is assigned a single physicist mentor who is responsible for all aspects of the assigned module including review of didactic material and providing a resident with hands‐on experience. Although it is not required that a mentor should also be a section chief at their institutions, it is beneficial when they are as they can efficiently incorporate the residents in the group in a structured way and include the residents in routine procedures and the QA schedule. A mentor can invite other members of the department to teach the resident specific tasks, given their expertise in specific areas.

*Research supervisors*: By its multi‐institutional nature, the program invites all faculty from the participating hospitals to mentor the residents and submit research proposals for a direct supervision of residents in their research year. Each year, residents select two projects from different institutions. Although there is a constraint that only one resident can be assigned to each of the two main institutions every year, the residents receive an abundance of projects to select from and residents are typically assigned their top choice.

#### 2.6.2 Treatment equipment and treatment planning systems

Another reason to form a multi‐institutional program is to offer the residents unparalleled training utilizing a wide range of clinical resources. There are two distinct advantages of a multi‐institutional program: (1) It offers the possibility to cover same procedures on different equipment (different brands of linear accelerators, CT simulators and different Treatment Planning and Record and Verify systems), and (2) it offers the possibility to include training in the modalities available only at one of the institutions, such as proton treatments, CyberKnife, MR‐linac, advanced image‐guided brachytherapy procedures, and adaptive treatments.

**FIGURE 2:**
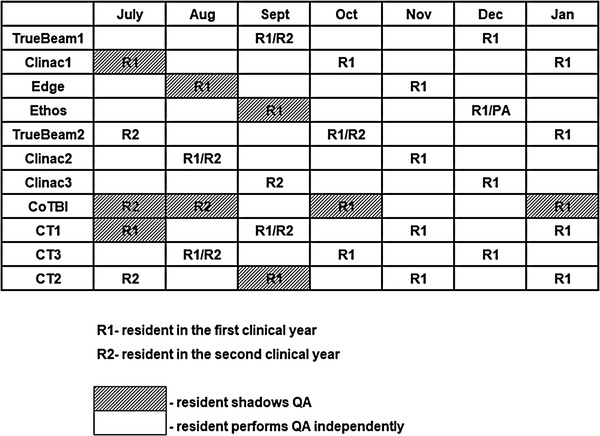
Example of machine quality assurance (QA) schedule of the residents at the Institution 1

The list of available resources combined between 3 institutions is presented in Appendix 1. To ensure that the residents receive hands‐on experience and sufficient proficiency with every available resource, a well‐organized schedule of training and coordination with clinical staff was developed. As an example, Figure 2 shows the residents’ machine QA schedule at the Institution 1 as part of the treatment equipment rotation. Similar schedule is developed at the Institution 2 for the same module, utilizing different machines. All other rotations are also required to develop a robust schedule before each resident starts the rotation.

### 2.7 Feedback mechanisms

For HMPRP to be successful, several elements of formal feedback mechanism were developed within the program. They include evaluations of residents by the mentors, evaluations of mentors and the program by the residents, and various meetings and discussions with the mentors in the program.

#### 2.7.1 Resident evaluations

Two commercially available web‐based residency management systems (initially, Typhon,^17^ Typhon Group, LLC, Metairie, LA and later New Innovations,^18^ Uniontown, OH) were adapted to collect various evaluations. Clinical and research mentors evaluate residents’ progress on a quarterly basis. A consistent set of questionnaires is used across all clinical training modules. All evaluations are discussed by the EXCOM, and as needed, by the Steering Committee. The mentors report on the residents’ progress, technical skills and knowledge, productivity and work ethic, interpersonal and communication skills, and professionalism. The residents evaluate the research year in terms of their satisfaction with the project, mentor's availability, research environment, and support. The residents also evaluate every clinical module quarterly. These evaluations address professionalism, availability and support of the clinical mentor, and frequency and quality of one‐on‐one meetings with mentors and quality of teaching. In addition, the residents are asked to comment on any time conflicts with other clinical modules, to offer suggestions for improvements of the modules, and to highlight any strengths of the rotation, access to didactic materials and availability of Program Director or Associate Program Directors.

*Oral assessments*: Quarterly oral assessments are used to evaluate each resident's progress during the clinical rotations. Faculty mentors from each module are present and ask questions relevant to the recent clinical experience of the resident. The Program Director and Associate Program Directors are also present to ensure consistency across three institutions. These examinations last two hours and serve as a metric of the clinical experience and knowledge of the resident, as well as preparation for the oral board exams. The Program Director summarizes the resident performance on the oral assessment in writing and reviews that feedback with the resident.

*Meetings with Program Directors*: The Program Director or an Associate Program Director meets with each resident monthly at their respective sites to evaluate and discuss the residents’ progress, exchange feedback, and discuss professional development. Any remarkable issues from these meetings are further reviewed at the EXCOM meetings.

In addition, regular surveys of the graduates are conducted by the program, and independent resident surveys are conducted by the GME office at the three institutions. Senior residents or recent program graduates are invited annually to an EXCOM meeting to provide in‐person feedback.

#### 2.7.2 Coordination of clinical rotations with multiple mentors

Several forms of communication with the mentors were established by (1) inviting mentors to select EXCOM meetings; (2) creating a clear path of communication between the mentors of the same modules from different institutions; (3) organizing annual mentors’ meetings. Although informal communication is also encouraged, the formal approach to organizing these meetings and discussions ensures appropriate communication flow and work toward specific goals.

### 2.8 Admissions

HMPRP recruits candidates for two positions each year. The main requirements for the prospective candidates are completion of a doctoral degree in physics, medical physics, or a closely related field and strong interest and potential in research, clinical innovation, and leadership. The entrance requirements for HMPRP do not include completion of a CAMPEP‐accredited program as the enrolled residents are able to fulfill all didactic requirements through the CAMPEP‐accredited Certificate Program embedded in HMPRP.

To support the efforts of the medical physics community in providing fair opportunities to the candidates and standardize the application process, HMPRP has been participating in MedPhys Match^19^ since 2014. Application reviews are initially performed by the members of EXCOM followed by a first round of short interviews with a selected group of candidates. Finalists then participate in formal interviews with a large group of faculty and staff from three institutions, as well as current residents. The finalists are also asked to give a short presentation.

Although challenges still exist with the match process itself,^20^ for HMPRP, the coordination of the interviews is especially difficult due to the number of candidates and multiple faculty members from different institutions. Up to 2019, the interviews were conducted over two consecutive days which was recognized as a burden on the candidates and the faculty. Starting in 2020, the interview process was moved to a virtual format. This change allows each of the candidates to spend only one day interviewing, although the faculty are still required to interview for two days due to the number of candidates. HMPRP, like many programs,^21^ is currently debating continuing virtual interviews as the cost‐benefit of residency interviews has been a concern.^22^

As interest in the residency from the candidates can be used as one of the metrics of success of the program, we routinely collect and analyze statistics on the applicants to our program and the feedback of the finalists. These surveys are conducted after the results of MedPhys Match are released to avoid any potential conflicts of interests.

### 2.9 Residents and graduates

An ultimate measure of success of the residency is the success of its residents and graduates. To characterize the productivity and achievements of the residents, we collected statistics on the number of publications by the residents, the awards that the residents have received, employment and ABR exams passing rate of the graduates. It should be noted that the official exam pass rate is characterized by verifying the board certification status through the ABR website, and available on the program's website.^14^ We can also estimate the initial pass rate for ABR Part 3 based on informal resident feedback and ABR data made available to program directors on request.

## 3 RESULTS

In this section, we describe the progress of HMPRP over 13 years since its inception and changes that were made along the way. Analysis of the residents’ productivity in the program and their success after the graduation is also presented.

### 3.1 HMPRP structure and governance

Although the general structure of governance of HMPRP did not change since 2009, the individuals serving on the committee did rotate over time. The role of EXCOM proved to be instrumental for continuous operation of the program throughout these changes, and it was especially important when the Program Directors changed or rotated between the institutions (5 times in 13 years). Additional changes to the governance structure involved adjustments to the frequency of the committees meetings. The frequency of EXCOM meetings was reduced from every 3 to every 4 weeks, whereas the frequency of the Steering Committee meetings was changed from annual to semiannual basis. These changes were implemented when the program's day‐to‐day operation reached its stable state. The current structure and the new frequency of the meetings were found optimal for program operations and improvements.

### 3.2 Curriculum

The curriculum and schedule of rotations underwent multiple modifications and adjustments, based on the residents’ and mentors’ feedback. Over the years, the main changes involved rearrangement of the rotations in the second clinical year to allow for the most efficient training. When new technologies are introduced to any of the clinics, new modules are developed to allow the residents to receive formal training on current topics. For example, a formal MR‐linac rotation was developed when an MR‐linac was installed at one of our clinics. The order and the duration of these modules has been adjusted to accommodate new topics and will continue to be adjusted due to residents’ feedback, changes in equipment and available procedures, and changes in CAMPEP standards. In addition, an extra treatment planning rotation was added to the second year curriculum to provide some flexibility in the first year curriculum and allow the resident additional time to complete the planning requirements.

### 3.3 Program statistics: faculty

In all, there are more than 60 Ph.D. and M.S. physics faculty at the three participating institutions. Many of these faculty serve as Clinical or Research mentors to the residents, and in some cases, both. Currently, there are 15 faculty members that are the core members of the program in their capacity and commitment as clinical mentors, as shown in Table 1. All faculty mentors are board certified.

On average, 11 research proposals are submitted by faculty members every year for consideration by the incoming residents. The topics of the research projects selected by the residents are listed in Table 2 and include studies on protons, radiomics, organ motion, brachytherapy, immunotherapy, and other topics. Seventeen different faculty mentors in total have directly supervised a resident in their research year during the past 13 years of the program. In some cases, two or more faculty members co‐supervised a resident's research project.

**TABLE 2:** Topics of research projects of Harvard Medical Physics Residency Program (HMPRP) residents in 2009–2021

Research topic	No. of projects
Proton therapy, including adaptive proton therapy	5
Proton imaging	4
Organ motion	4
Radiomics	3
Image‐guided brachytherapy	3
Modeling immune response in combination with radiotherapy	2
Imaging	1
Novel detector design	1
Radiobiology	1
Nanoparticles in radiation therapy	1
FLASH radiotherapy	1
Total	26

When 2 research faculty members (directly supervising the residents during the research year) and 15 clinical mentors are included in the calculation, the faculty to resident ratio is 17:6 or 2.8:1.

### 3.4 Clinical resources

Installation of new equipment and upgrades are common at our institutions. Since 2009, seven new treatment machines were installed (marked with * in Appendix 1), and multiple linear accelerators and CT simulators were replaced. All residents enrolled in the program at the time of a new installations have participated in acceptance and commissioning of the new equipment. When new technology was introduced in clinic, the training curriculum was adjusted to accommodate residents’ training on the new modalities.

### 3.5 Program statistics: enrollment, productivity and achievements of residents, and graduates

Every year, without exception, HMPRP admitted two residents. On average, our program had 68 applicants per year with the lowest number of applicants (36) in 2011 and the highest (132) in 2016, Table 3. Since 2017, the number of applicants remained stable, fluctuating between 56 and 67, and averaging 62 applicants per year.

**TABLE 3:** Program statistics as of May 2022

Resident class	Applicants	Admitted residents	Graduated residents	Board certified	Employment—clinical/academic	Employment—clinical/nonacademic
2009–2012	71	2	2	2	2	0
2010–2013	64	2	2	2	2	0
2011–2014	36	2	1^a^	1	1	0
2012–2015	56	2	2	2	2	0
2013–2016	63	2	2	2	2	0
2014–2017	49	2	2	2	2	0
2015–2018	108	2	2	2	2	0
2016–2019	132	2	2	2	1	1
2017–2020	60	2	2	2	2	0
2018–2021	66	2	2	2	2	0
2019–2022	62	2	0	n/a	n/a	n/a
2020–2023	56	2	0	n/a	n/a	n/a
2021–2024	67	2	0	n/a	n/a	n/a
2022–2025	59	0	0	n/a	n/a	n/a
Total	949	26	19	19	18	1
*Average*	*68*	*2*				
*Min*	*36*	*2*				
*Max*	*132*	*2*				

^a^One resident did not complete the program for medical reasons.

Out of 26 admitted residents, 12 (46%) residents entered the program after completing a CAMPEP‐accredited PhD or Certificate Program (3 out of 12 residents have completed our own Certificate Program before entering the residency), whereas 14 (54%) residents needed to take all or some didactic courses to fulfill the CAMPEP course requirements. All of these residents were able to complete the required coursework within our Certificate Program before graduating from the residency.

All residents spent their first year working on research projects as summarized in Table 2. The resident publication record is summarized in Table 4. As of May 2022, the HMPRP residents authored or co‐authored 71 publications, averaging 5.5 publications per year, and 3 publications per resident (note that only 24 out of 26 residents ever enrolled in HMPRP as of May 2022 are included in this count, since 2 first year residents are still preparing manuscripts for publications). Overall, 6 out of 71 publications (8.5%) received recognition, see Table 4. In addition, residents presented at numerous local, national, and international conferences, on average 9 abstracts per resident over the course of the residency. The residents also received multiple awards, including nine residents selected in a very competitive SCAMP Program,^23^ see Table 5.

**TABLE 4:** Publications authored by Harvard Medical Physics Residency Program (HMPRP) residents as of May 2022

Year	No. of publications in a given year	Highlighted publications
2010	2	
2011	4	
2012	6	Editor's choice article
2013	4	
2014	3	One of PMB highlights of 2014 collection
2015	5	Editor's pick article
2016	6	
2017	10	
2018	3	Featured article
2019	5	
2020	8	
2021	12	Invited submission: focus on early career researchers in physics and medicine and biology
2022	3	Winner 2022 Roberts Prize for best paper in physics and medicine and biology
Total	71	

**TABLE 5:** Awards received by Harvard Medical Physics Residency Program (HMPRP) residents as of May 2022

Awards	No. of awards	Years
Roberts Prize for Best Paper in PMB	1	2021
Young Investigator Symposium Winner, New England Chapter of the AAPM (NEAAPM)	2	2021, 2022
Particle Therapy Cooperative Group (PTCOG) Travel Fellowship	1	2020
AAPM Conference Best in Physics Abstract	1	2019
AAPM Jack Fowler Junior Investigator Award	2	2019, 2022
AAPM Best Award Travel Fellowship	1	2019
American Brachytherapy Society Conference Travel Award	1	2019
Particle Therapy Cooperative Group (PTCOG) Michael Goitein best abstract award	1	2019
Winter Institute of Medical Physics Early Career Scholarship Award	1	2019
Winner, AAPM Research Seed Funding Grant	1	2022
Residents selected to the AAPM Science Council Associates Mentorship Program (SCAMP)	9	2015(2), 2017(2), 2019, 2021(2), 2022(2)

Abbreviation: AAPM, American Association of Physics in Medicine.

As of May 2022, all eligible graduates have passed Part 3 of the ABR exam. Further, based on informal resident feedback and ABR data made available to program directors on request, we estimate that the initial pass rate for the ABR Part 3 exam is approximately 90%. All graduates found employment as medical physicists and 18 out of 19 graduates obtained jobs in major academic centers, see Table 3.

## 4 DISCUSSION

In this report we describe our experience creating and managing a multi‐institutional clinical and research medical physics residency program. To the best of our knowledge, this is the only multi‐institutional medical physics training program in the United States. The program was built upon a long‐standing strength of several academic hospitals in close geographical proximity to each other with an idea of creating a nourishing environment for training future medical physicists with research and leadership interests.

There are multiple advantages to creating such a program. By combining the resources of several hospitals, the residents are offered access to a wide variety of equipment, clinical procedures, and faculty expertise. In addition to providing strong clinical training, this also allows a wide range of research topics to the residents and even the opportunity to pivot during their research should the residents encounter unanticipated barriers. The 3‐year nature of the program allows outstanding candidates without a degree in medical physics to take didactic courses and fulfill CAMPEP course requirements while in residency. In addition, the combined program offers multiple opportunities for collaboration and educational activities, such as seminars and lectures.

Due to all of the aforementioned factors, HMPRP has been very successful. It solidified quickly after its formation in 2009 and has received CAMPEP accreditation followed by two successful required reaccreditations at 5‐year intervals. Judging by the number of applicants, HMPRP is positively viewed among prospective residents. The number of applicants has been steady since 2017 and currently averages at around 62. The admitted residents had excellent performance, completing all required clinical training and producing excellent research outcomes, resulting in multiple publications and awards. Although more than half (54%) of the candidates entered the residency without formal CAMPEP degree, the HMPRP residents were able to complete required course work before graduating from residency. The residency also gained a solid reputation among prospective employers. Most of the graduates were hired and are currently employed in large academic centers. Perhaps most importantly, the residents receive solid clinical training combined with research experience as evidenced by the fact that all graduates achieved ABR certification and remain employed at academic centers (one graduate changed fields to join a different but extremely competitive program outside of the field).

It is also important to recognize that managing a program with a wide variety of resources and faculty, especially those from different institutions, poses significant logistical challenges.

Based on the progress of our program over 13 years, the following factors are crucial for successful multi‐institutional program coordination and management:

1. administrative and financial support from different hospitals from the time of formation of the program,
2. a structure with built‐in collaboration and coordination between the institutions,
3. formal curriculum and formal evaluation of the residents by multi‐institutional faculty,
4. comprehensive structure of governance including sole Program Director for overall leadership, Associate Program Directors for direct oversight at respective institutions, EXCOM for program coordination and exchange of information between Director and Associate Directors, and Steering Committee for high‐level oversight,
5. comprehensive feedback mechanism, frequent review of the program, and adjustments as necessary.

There are several important considerations for programs that consider starting a multi‐institutional residency. The first consideration is recognizing that such a program requires much stronger coordination of resources, solid planning, and monitoring that may be needed for a program in a single institution. A comprehensive leadership structure is critical for the program's success. Another consideration is that the program should be structured in a way to allow for frequent adjustments, changes of individual mentors and members of the leadership team as well as changes in available resources. Some level of flexibility in the program's structure and curriculum should be built‐in to allow for the smooth operation of the program when adjustments are necessary. This should be considered when creating a well‐planned and optimized curriculum and schedule of modules. To allow for this flexibility, our program has built‐in an extra treatment planning rotation in the end of the clinical training to allow the residents to catch up with the training in the case of unexpected scheduling rearrangements. If research year is part of a program, it is critical to set the right expectations for both the residents and the research mentors. Frequent communication between the research mentors and the Program Directors should be an important part of the program operation. Finally, a complicated structure of the program creates substantial administrative burden. Appropriate resources need to be allocated at all institutions to provide administrative support and coordination and substantial time should be allocated to the Program Directors and Associate Program Directors not only to lead the program at each individual institution but also for frequent communications among different centers.

It also should be recognized that although a 3‐year multi‐institutional program has distinct advantages, it may not be appealing to all residency candidates and should be viewed as one of several options that are available for incoming residents. Although some of the candidates consider an addition of extra year to their training an advantage and an opportunity to get introduced to the medical physics and develop productive research, for others a 2‐year program is more attractive as a faster career track. Some candidates may also prefer to start with a postdoctoral position before entering the residency. This path provides some flexibility of deciding on the next step of the career path after the postdoctoral training, whereas a 3‐year residency program requires a long‐term commitment from the beginning. In turn, the residents enrolled in a 3‐year program have a benefit of being integrated in the residency sooner, building ties with the current residents and mentors and becoming familiar with the clinical aspects of the field through research. In our experience, emphasizing these differences in programs is an important part of the interview to make sure that candidates develop a good understanding of their options and make the right decision about their future career.

As our program continues to grow, it will likely face new challenges. The current structure of the program management and operation is designed to provide a solid foundation for addressing future challenges and making the appropriate adjustments to the program. Our experience in forming such program and optimizing its structure and operation may help other residencies to consider if a multi‐institutional program may be the right choice for them.

## 5 CONCLUSIONS

A multi‐institutional medical physics residency program can be successfully formed and managed. With a collaborative administrative structure, the program creates an environment for high‐quality clinical training of the residents and high productivity in research. The main advantage of this program is access to a wide variety of resources. The main challenge is creating a robust structure for efficient management of multiple resources at different locations. This report may provide valuable information to centers considering starting a multi‐institutional residency program.

## AUTHOR CONTRIBUTIONS

All authors contributed equally to this article.

## CONFLICT OF INTEREST

No conflict of interest.

## APPENDIX 1

### 1.1

Clinical resources combined among three institutions available for residents training (these numbers represent equipment on the main campuses only and do not include the network locations, where residents also spend time during residency; note: the * indicates equipment installed after 2009)

- Seventeen linacs, a mix of Varian (Varian Medical Systems, Palo Alto, CA) and Elekta (Elekta, Crawley, UK) brands, including the following:
  ◦ dedicated stereotactic linear accelerators, Edge (Varian) (*)
  ◦ two Ethos adaptive treatment units (Varian) (*)
- CyberKnife unit (M6 FIM, Accuray Inc, Sunnyvale, CA)
- MRI‐guided linac (MRIdian, ViewRay Inc, Mountain View, CA) (*)
- Proton cyclotron machine with three treatment rooms (IBA, Louvain‐la‐Neuve, Belgium)
- Compact synchrotron proton machine (Protom, Flower Mound, TX) (*)
- Several CT simulators from GE (Waukesha, WI), Siemens, and Canon Aquilion LB
- MR simulator (3T MAGNETOM Vida, Siemens) (*)
- Dedicated IORT electron linear accelerator in the OR suite
- Dedicated Cobalt‐60 TBI unit (GammaBeam 500, Best Theratronics, Ottawa, CA) (*)
- Linac‐based TBI program
- Comprehensive HDR program, including Oncentra Brachy treatment planning system (Elekta Brachytherapy)
- Image‐guided HDR suite with a dedicated CT and MR
- Three different treatment planning platforms, including the following:
  ◦ Eclipse (Varian Medical Systems, Palo Alto, CA)
  ◦ RayStation (RaySearch Laboratories AB, Stockholm, Sweden)
  ◦ Pinnacle (Philips Radiation Oncology Systems, Fitchburg, WI)
- Two different R&V systems, including the following:
  ◦ ARIA (Varian Medical Systems, Palo Alto, CA)
  ◦ Mosaiq (Elekta, Crawley, UK)
